# When is Sentinel Lymph Node Biopsy Useful in Ductal Carcinoma In Situ? The Experience at a Latin American Cancer Center

**DOI:** 10.7759/cureus.16134

**Published:** 2021-07-03

**Authors:** Sandra E Diaz Casas, Wilmar A Serrano Muñoz, Nelson A Buelvas Gómez, Ana M Osorio Ruiz, Javier Ángel Aristizábal, Luis H Guzmán Abisaab, Mauricio Garcia Mora, Carlos Lehmann Mosquera, Sergio Cervera-Bonilla, Ricardo Sanchez Pedraza

**Affiliations:** 1 Breast and Soft Tissue Surgery, Instituto Nacional de Cancerología, Bogotá D.C, COL; 2 Breast Surgery, Instituto Nacional de Cancerología, Bogotá D.C, COL; 3 Epidemiology and Public Health, Instituto Nacional de Cancerología, Bogotá D.C, COL

**Keywords:** breast carcinoma in situ, ductal carcinoma in situ, sentinel node biopsy, axillary lymph nodes, lymph node metastases

## Abstract

Introduction

Ductal carcinoma in situ (DCIS) accounts for 15% of breast cancers. Surgery is the main treatment, and the use of sentinel node biopsy (SLNB) is restricted to patients at risk of infiltration, which is estimated to be around 26%.

Materials and methods

Aimed at evaluating the benefit of SLNB in patients with DCIS at the Breast and Soft Tissue Functional Unit of the National Cancer Institute (INC for its initials in Spanish), a descriptive observational study of a retrospective cases series was conducted between August 1, 2013, and September 30, 2018.

Results

A total of 40 patients with a median age of 57 years were included in the study; 62.5% of them underwent mastectomy with SLNB, and the remaining 37.5% underwent conservative surgery with SLNB. 100% of sentinel nodes were identified, by using lymphoscintigraphy in 95%. Sentinel node was positive in four patients (10%), three of whom had infiltration in the surgical specimen reported. With a follow-up of 49 months, only one patient had a local relapse. None of the patients had axillary or distant recurrence.

Conclusions

SLNB in DCIS should be limited to patients with risk factors for infiltration (tumor size greater than 3 cm, comedo-type histology, and high-grade DCIS), and patients with an indication for mastectomy. Its percentage of complications is low, and a high identification percentage in surgical groups with adequate training.

## Introduction

Breast cancer ranks first in incidence in women in the world. According to Global Cancer Incidence, Mortality, and Prevalence (GLOBOCAN) 2020 data, 2,261,419 new cases were diagnosed, which account for 11.7% of all cancers, and 684,996 deaths were recorded for both genders. In Colombia, breast cancer ranks first in incidence and third in mortality; for the same year 2020, a total of 15,509 new cases were reported, which accounts for 13.7% of the total cancers reported in the country [1]. Currently, ductal carcinoma in situ (DCIS) represents 15% of newly diagnosed breast cancer, and in most cases, diagnosis is incidentally made by finding microcalcifications during a screening mammogram [2].

The term breast carcinoma in situ is used to describe lesions of abnormal epithelial cells confined to the lobes or ducts of the breast, but similar in appearance to invasive carcinoma cells, without exceeding the basal membrane. It was argued for a long time that these cells could invade the adjacent breast stroma and eventually progress to invasive cancer. However, carcinoma in situ does not fully express several of the characteristics of invasive carcinoma, and the molecular changes involved in progression to invasive cancer do not always occur. [3,4]

Overall prognosis is good, with specific mortality of 3% at 15 years. In follow-up studies of patients with DCIS who did not undergo surgical resection, it was found that between 20% and 53% of patients were diagnosed with invasive breast cancer within the next 10 years, finding that invasive local recurrences can lead to distant metastases in 12% to 15% of cases [2,5].

DCIS treatment is essentially surgical, ranging from conservative surgery to radical surgery depending on lesion extent. The use of sentinel node biopsy (SLNB) is restricted to patients at risk of infiltration, which is estimated to be around 26% [6]. Although SLNB is a minor procedure with low morbidity rates, there is a 6% risk of developing lymphedema [7]. The meta-analysis carried out by Brennan et al. shows that the preoperative variables that significantly increase the risk of infiltration in DCIS are: use of thinner biopsy needles, tumors larger than 20 mm, a palpable lesion, and mammography categorized as Breast Imaging Reporting and Database System (BI-RADS) IV or V [6]. The literature reports presence of metastatic positive nodes in patients with DCIS ranging from 2% to 15% [8-10].

Current guidelines recommend SLNB in patients with DCIS who require a mastectomy since no lymphatic vessels are left after radical surgery (they are excised in bloc with the rest of the fibroglandular breast tissue), and therefore an SLNB could not be performed in a subsequent surgery in case that pathology reports infiltration [11]. Other indications for SLNB in DCIS include tumor high suspicion of infiltration (tumors greater than 3 cm or a high histological grade, and presence of comedo necrosis pattern) [12], all of these factors increasing the rate of underestimating infiltrative disease. Based on these indications and despite the low probability of obtaining a positive SLN, SLNB in DCIS has become an increasingly common practice, so much so that it was performed in 19% of patients with DCIS undergoing breast-conservative surgery and in 63% of patients with DCIS undergoing mastectomy in the USA in 2012. [13].

Regarding the usefulness of SLNB in patients with DCIS who are undergoing breast-conserving surgery, two large studies have been recently reported. The first one was carried out by El Hage Chehade et al., who in 2017 published a meta-analysis evaluating 9,803 patients from 48 studies, recommending that SLNB could be safely omitted in DCIS smaller than 2 cm with a high histological grade, or in DCIS greater than 2 cm with low or intermediate histological grades [14]. In the second one in 2019, Hung et al. showed a study based on a SEER population with 1992 patients, with older adults in the age range of 67 and 94 years who were taken to breast conservative surgery (BCS) and SLNB, with no impact observed on locoregional recurrence or in general survival, suggesting the little benefit of SLNB in older patients with DCIS who undergo BCS [15].

The main objective of this work is to evaluate sentinel node performance in patients with DCIS who underwent surgical treatment at the Breast and Soft Tissue Functional Unit of the National Cancer Institute (INC for its initials in Spanish) between September 1, 2013 and August 31, 2018; percentage and type of complications derived from this surgical technique and tumor relapse.

## Materials and methods

A retrospective case series descriptive observational study was carried out between September 1, 2013 and August 31, 2018, which included all patients with a diagnosis of DCIS registered in the database of the Functional Breast and Soft Tissue Unit of the Nacional Cancer Institute, who underwent surgical management of the primary tumor and SLNB. The information was collected from data recorded in the INC's SAP® electronic medical records system (SAP AG, Walldorf, Germany). Information collection was recorded in a format designed with the study variables, which included demographic, histopathological, treatment, and follow-up information. Patient data were collected and recorded by three of the authors in an electronic database based on the REDCapTM platform (Vanderbilt University, Nashville, TN); data were later reviewed by the authors, and their quality was supervised by the monitoring center of INC research department. The Functional Unit has established to follow up the patients with DCIS every three months during the first two years and every six months between the second and fifth year and finally every year after the fifth year. The mammography is performed annually. In cases with follow-up loss, the patient or her relatives were contacted by telephone to verify whether or not the outcome related to disease-free survival had occurred.

Patients with a histological diagnosis of DCIS, who underwent surgical management of the primary tumor (radical surgery or BCS) and SLNB by one of the specialists of the Functional Breast and Soft Tissue Unit of the INC during the study period were included. Patients not undergoing axillary intervention were excluded, as well as patients with a diagnosis of infiltrating carcinoma in the pathology review, and those who were not managed at the institution.

Variables were analyzed by using conventional methods of descriptive statistics; categorical variables were summarized using absolute frequencies and percentages, and continuous variables were summarized using medians and interquartile ranges (IQR).

The statistical analysis of the information was carried out in the STATA 15.0 software licensed for the National Cancer Institute.

## Results

**Figure 1:**
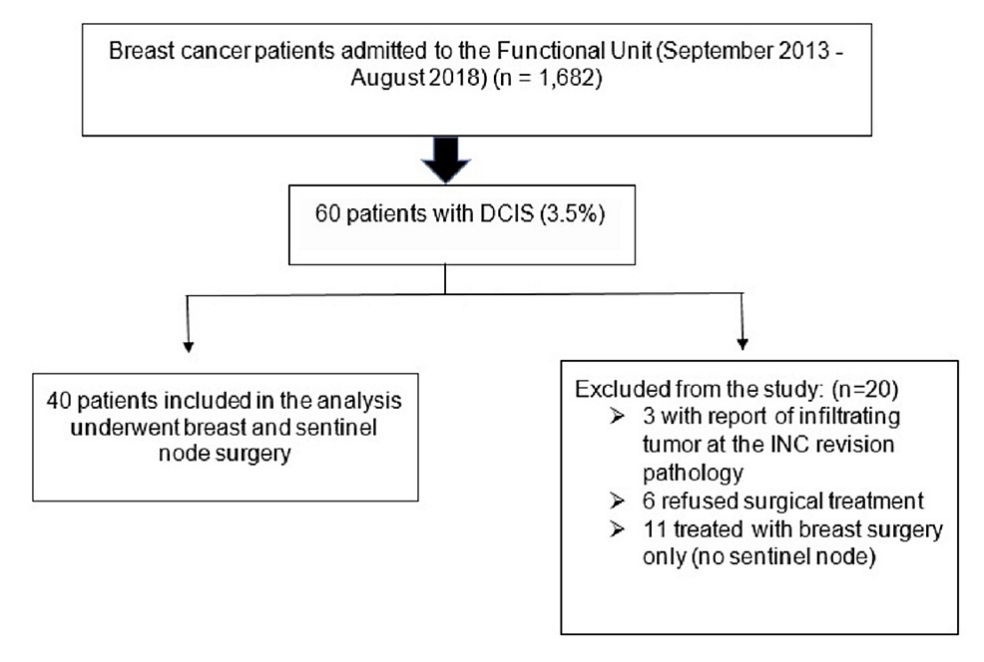
Inclusion criteria

A total of 1,682 patients diagnosed with breast cancer were admitted to the INC between September 1, 2013 and August 31, 2018; of these, 1,336 patients (79.4%) had invasive cancer in early and locally advanced stages, 286 (17%) were in metastatic stage (IV), and 60 patients (3.5%) had to DCIS, which were candidates to be included in present study; 20 of them were excluded for the following reasons: three patients had an infiltrating component in the INC revision pathology, six did not accept surgical treatment, and 11 patients were treated only with breast surgery and no SLNB was performed. So, of these 60 patients, 40 met the study inclusion criteria and were entered for analysis (Figure 1).

The median age was 57 years (IQR = 13 years), from 37 to 77 years. All patients were female. On physical examination, the tumor was palpable in 70% (n = 28) of patients. In the biopsy pathology report, only 47.5% (n = 19) had a single histological type, and 53.5% (n = 21) had a combination of 2 and 3 histological types. The predominant histological subtype was comedo in 57.5% (n = 23); 65% (n = 26) of the patients had histological grade III, and 67.5% (n = 27) were hormone receptor (HR) positive. Table 1 shows the demographic and clinical characteristics of the patients.

**Table 1:** Demographic and clinical characteristics of patients included in the study *21 Patients presented 2 or 3 histological types in the biopsy; the table reports the total number for each histological type, regardless of combinations.

Characteristic	Number (n=40)	Percentage (%)
Age: median (IQR)	57.5 (37-77)	
Health scheme	n	%
Contributory	24	60
Subsidized	16	40
Presence of palpable tumor	n	%
Palpable tumor	28	70
Non-palpable tumor	12	30
Histological type (biopsy)*	n	%
Comedo	23	57.5
Solid	16	40
Papillary	1	2.5
Micropapillary	1	2.5
Cribriform	12	30
No data	6	15
Histological grade	n	%
1	1	2.5
2	11	27.5
3	26	65
No data	2	5
Hormone receptors	n	%
Negative	10	25
Positive	27	67.5
No data	3	7.5
Ki 67	n	%
≤20%	3	7.5
≥21%	6	15
No data	31	77.5

Regarding surgical treatment of the primary tumor, 62.5% (n = 25) of the patients underwent mastectomy, and 37.5% (n = 15) underwent BCS. Indications for performing a SLNB were: mastectomy performed due to poor breast-tumor relationship and multicentricity in 60% (n = 24); suspected infiltration in the initial biopsy 17.5% (n = 7); ductal carcinoma in comedo-type situ in 55% (n = 22); high histological grade in 60% (n = 24) of cases; and tumor size greater than 3 cm in one patient. Most of the patients had more than one indication 65% (n = 26) for SLNB.

Sentinel node identification was achieved in 100% of cases, using lymphoscintigraphy in 38 patients (95%), and dual technique (lymphoscintigraphy + methylene blue) in two patients (5%). Distribution of the number of sentinel and non-sentinel nodes obtained during the procedure are summarized in Table 2.

**Table 2:** Type of surgical treatment received by the patients of the study

Characteristic	Percentage (%)	Number
Type of surgical treatment of primary tumor		
Simple mastectomy	62.5	25
Conservative surgery	37.5	15
Sentinel node identification technique		
Lymphoscintigraphy	95	38
Dual technique	5	2
Sentinel node identification percentage	100	40
Number of resected sentinel nodes		
1	55	22
2	32.5	13
3 or more	12.5	5
Patients with resected non-sentinel nodes.	32.5	13
Number of resected non-sentinel nodes		
1	69.23	9
2	7.69	1
3 or more	23.08	3

Regarding the pathology report of the surgical specimen, 7.5% (n = 3) of patients had margins <2 mm, and 12.5% (n = 5) had positive margins. Of the total eight patients with positive and close borders, five were taken to second surgery for margin clearance, mastectomy was made in one patient, and margin clearance was not performed in two patients due to non-acceptance of the procedure.

Sentinel node was positive in 10% (n = 4) of patients, two of these were macrometastasis. All had palpable lesions of high histological grade; HR positive and infiltrating tumor in the surgical specimen was found in three of them.

None of the non-sentinel nodes assessed presented metastasis. As for patients with positive sentinel lymph nodes, axillary management with axillary dissection was performed in two of them, who were patients whose SLNB pathology reported macrometastasis; axillary dissection was omitted in the other two patients (Table 3).

**Table 3:** Anatomopathological features of the surgical specimen of the primary tumor and the sentinel node * 25 pathologies report more than one histological type.

Characteristic	Number ( n)	Percentage (%)
Tumor size		
<1cm	7	17,5
1-2 cm	14	35
>2cm	15	37,5
No data	4	10
Mean primary tumor size = 2.38cm	(IQR: 2,4)	
In situ tumor histological type*	n	%
Comedo	20	50
Solid	14	35
Papillary	4	10
Micropapillary	3	7.5
Cribriform	14	35
No data	8	20
Infiltration type		
In situ	19	47.5
Presence of infiltrating tumor	21	52.5
Resection edges	n	%
Free	32	80
Close to <2mm	3	7.5
Positive	5	12.5
Sentinel node positive patients	4	10
Type of sentinel node metastatic involvement	n	%
Single tumor cells or small clusters	1	2
Micrometastases	1	2
Macrometastases	2	5

53.5% (n = 21) of patients had an infiltrating tumor in the final pathology report, 10 of them with tumor size greater than 2 cm, 11 patients with comedo histological type, and 13 patients with histological grade III.

Only one patient (2.5%) had a complication associated with sentinel node surgery, related to a seroma that was treated with drainage in the outpatient clinic. Regarding primary tumor surgery, four patients presented seroma, two of them associated with superficial surgical site infection (SSI).

80% (34) of patients received adjuvant treatment: 27.5% (n = 11) of them received chemotherapy because they have an infiltrating tumor in the final pathology report; 42.5% (n=17) radiotherapy and 67.5% (n = 27) hormone therapy.

With a 49-month follow-up, only one patient presented local recurrence; this was a patient with comedo-type DCIS, without the presence of an infiltrating tumor, a pathology report with a near margin that did not allow the second surgery for margin clearance. None of the patients in the series had an axillary or distant recurrence.

## Discussion

DCIS is a lesion confined to the breast ducts without invading the adjacent stroma, reason why these lesions incompletely express the characteristics of invasive cancer, therefore making the SLNB technique to be currently restricted to precise indications, which include: tumor size greater than 3 cm, patients undergoing mastectomy, biopsy report with a comedo-type lesion, and high histological grade. However, there is still controversy as to whether it should always be performed or restricted to the already described indications.

All patients in our study had one or more of these indications. 100% of sentinel lymph nodes were found by using the lymphoscintigraphy and dual technique, and a 10% (n = 4) positivity of the sentinel node. This is similar to what Price et al. in USA reported, who found positivity for SLNB in 10.4% of patients undergoing mastectomy plus SLN, and to what Al-Ameer et al. in Saudi Arabia reported, who obtained positivity in two out of 20 patients, which amounts to 10% of the study population [16,17]. Van Roozendaal et al. in the Netherlands also reported 9.3% sentinel node positivity, with 3.8% positivity with isolated cells, 3% micrometastasis, and 2.5% macrometastasis, results similar to those of our study [10].

Regarding the sentinel node positivity rates in Latin American countries, we find similarities in an experience by Ruvalcaba et al. in Mexico, who report positivity of 8% (n = 4); however, it is striking that they mainly used the dual technique (lymphoscintigraphy + patent blue) in 82% of the cases in their study, as opposed to our experience, in which the sentinel node was identified in 95% of cases by lymphoscintigraphy, and only 5% by using the dual technique [18].

We found notable differences in terms of results in the Italian experience led by Intra (2003), where 223 patients with DCIS underwent SLNB, obtaining a positivity of only 3.1%; this may be due to the fact that all SLNBs in this study were performed at the same surgical time of the primary tumor, while in the study by Intra et al, performance was delayed in 14.8% of cases until a pathology report of the primary tumor was obtained; another reason for this discrepancy may be the entire sample [8]. We also found differences with the experience by Heymans et al. who reported 3.1% positivity of the sentinel node, probably due to the fact that they performed SLNB only in 66.7% of patients diagnosed with DCIS [9]. And in a Venezuelan experience, where they did not find positivity for SLNB in any patient, despite having a total of 64 patients with a diagnosis of DCIS and a sentinel node identification rate of 95.3%; the study population had the infiltrating component underestimated because they used a vacuum cutting system to diagnose the primary tumor [19]. However, it must be clarified that these values are within the ranges reported in the literature, which range between 1% and 15%.

The sentinel node procedure has gained strength for axillary staging in recent decades, as it represents a reduction in the risk of complications, which translates into better postoperative evolution and better quality of life for patients.

Numerous studies have compared the morbidity associated with the sentinel node procedure vs axillary lymph node dissection (ALND).; in the NSABP B-32 study, the authors found differences in morbidity associated with a three-year follow-up, favoring SLNB in the following aspects: deficit in shoulder abduction > 10% (41% SLNB vs 75% ALND); lymphedema > 10% (8% SLNB vs 14% ALND); sensitivity alteration in the inner face of the arm (23% SLNB vs 49% ALND) [20]. In the prospective study by Langer in 2007, with a population of 651 patients, they found statistically significant differences in terms of morbidity in favor of the sentinel node (35.8% vs 66.2%. P = <0.0001), unlike the NSABP B32; in this study, they also reported immediate postoperative complications such as seroma 1.8%, hematoma 1.8%, and wound infection 0.9%, complications that we studied in this report, finding only one patient with a seroma associated with the SLNB procedure [21].

DCIS locoregional relapse has been reported in 32.4% of patients managed with breast-conserving surgery, and in 12.6% in patients undergoing breast-conserving surgery plus radiotherapy with 84 months follow-up; relapse percentages are directly related to the presence of associated infiltrating carcinoma and to the positivity of the edges reported in the definitive pathological anatomy, which is associated with identification of residual DCIS in 40% to 82% of resection samples, correlating with margin width: 41% at <1 mm, 31% at 1-2 mm, and 0% with ≥2 mm clearance [22].

In this study, with 49 months of follow-up, only one patient presented local recurrence, with the particularity that it was a comedo-type tumor, without the presence of an infiltrating tumor and with a near border, which did not allow the margin to be expanded. This discrepancy in the percentages can be explained by the shorter follow-up time in our study compared to the studies reported in the literature, but it corroborates the higher probability of relapse in cases with positive borders.

The main limitations of our work are those inherent to a retrospective study of a single institution.

## Conclusions

In this cohort, the high histological grade, the presence of comedo necrosis and palpable tumors were related to metastasis of the sentinel node in patients with DCIS. For these reasons, the SLNB in DCIS should be limited to patients with risk factors for infiltration (tumor size greater than 3 cm, architectural pattern comedo necrosis, and high grade), and in patients with indication for mastectomy. This procedure has a low percentage of complications and a high percentage of identification in surgical groups with proper training.
